# Nanoparticles Supported on Sub‐Nanometer Oxide Films: Scaling Model Systems to Bulk Materials

**DOI:** 10.1002/anie.202015138

**Published:** 2021-01-28

**Authors:** Kevin Ament, Nicolas Köwitsch, Dianwei Hou, Thomas Götsch, Jutta Kröhnert, Christopher J. Heard, Annette Trunschke, Thomas Lunkenbein, Marc Armbrüster, Josef Breu

**Affiliations:** ^1^ Bavarian Polymer Institute and Department of Chemistry University of Bayreuth Universitätsstraße 30 95447 Bayreuth Germany; ^2^ Faculty of Natural Sciences Institute of Chemistry Materials for Innovative Energy Concepts Chemnitz University of Technology Straße der Nationen 62 09111 Chemnitz Germany; ^3^ Department of Physical and Macromolecular Chemistry Charles University Hlavova 8 128 00 Prague 2 Czech Republic; ^4^ Department of Inorganic Chemistry Fritz-Haber-Institut der Max-Planck-Gesellschaft Faradayweg 4–6 14195 Berlin Germany

**Keywords:** Clay, CO oxidation, Metal support interaction, Palladium, Ultrathin oxide layer

## Abstract

Ultrathin layers of oxides deposited on atomically flat metal surfaces have been shown to significantly influence the electronic structure of the underlying metal, which in turn alters the catalytic performance. Upscaling of the specifically designed architectures as required for technical utilization of the effect has yet not been achieved. Here, we apply liquid crystalline phases of fluorohectorite nanosheets to fabricate such architectures in bulk. Synthetic sodium fluorohectorite, a layered silicate, when immersed into water spontaneously and repulsively swells to produce nematic suspensions of individual negatively charged nanosheets separated to more than 60 nm, while retaining parallel orientation. Into these galleries oppositely charged palladium nanoparticles were intercalated whereupon the galleries collapse. Individual and separated Pd nanoparticles were thus captured and sandwiched between nanosheets. As suggested by the model systems, the resulting catalyst performed better in the oxidation of carbon monoxide than the same Pd nanoparticles supported on external surfaces of hectorite or on a conventional Al_2_O_3_ support. XPS confirmed a shift of Pd 3d electrons to higher energies upon coverage of Pd nanoparticles with nanosheets to which we attribute the improved catalytic performance. DFT calculations showed increasing positive charge on Pd weakened CO adsorption and this way damped CO poisoning.

## Graphical abstract

Palladium nanoparticles were partially covered by sub‐nanometer thick layers of hectorite. Due to electronic interactions between the nanoparticles and the ultrathin layers the nanoparticles were positively charged. This in turn improved the performance for the oxidation of carbon monoxide.

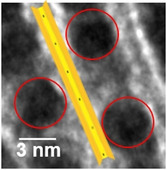

## Introduction

Many nanoparticulate catalysts are prepared by wet impregnation on an oxidic support. The oxidic surface is often regarded as an “inert” support assuring stabilization and dispersion while hampering Ostwald ripening.^[1]^ Recent results show, however, that the right choice of support can have significant influence on the selectivity and activity of catalysts.^[2]^ In particular, the so called electronic‐metal‐support interaction (EMSI) was shown to alter the catalytic performance of catalysts by electronic interaction between support and metal.^[3]^ In the past years, model systems were applied, which are based on ultrathin oxidic films deposited on atomically flat metal surfaces, to study EMSI.[^2a^, ^4^] A modification of the work function of the metal was observed when thin oxide films were deposited on flat metal surfaces.^[5]^ This phenomenon can be attributed either to charge transfer between metal and support, electrostatic, or compression effects.[^5b^, ^6^] For model catalysts composed of Pt^[7]^ or Ir^[8]^ clusters deposited on CeO_2_ films, a charge transfer from the noble metal to the oxide was observed resulting in a positively charged metal cluster.

**Scheme 1:**
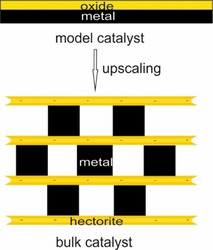
Upscaling of a model architecture consisting of a single thin oxide layer (<1 nm) on bulk metal to a mesostructured catalyst with large accessible active area by exploiting the layered silicate hectorite as thin separator.

Such model catalysts helped to greatly deepen our understanding of the performance of real catalysts under working conditions. While model films can be fabricated with utmost control (Scheme 1),^[9]^ synthesis protocols for sub‐nanometer oxidic supports as required for bulk‐scale materials are lacking.

Negatively charged layered materials such as clays have been explored as supports for nanoparticles and their catalytic performance has been tested to some extent.^[10]^ Taking advantage of the cation exchange capacity, desired cations have been introduced on and/or between the silicate layers followed by reduction (e.g. Pd, Cu, Ru)^[11]^ or precipitation (e.g. CdS)^[12]^ to obtain the final nanoparticulate catalysts. Typical cation exchange capacities for natural clays of <100 mmol/100 g^[10]^ limit the loading (e.g. ≈6 wt % Pd) that can be obtained via the cation exchange route corresponding to as little as one particle per 1500 nm^2^ (assuming 3.5 nm sized particles). As natural clays typically come in lateral sizes smaller than 200 nm, the very few nanoparticles produced this way preferentially end up at external surfaces as suggested by *Dékány* et al.^[13]^ and as indicated by insignificant shifts of the basal spacing.

To achieve a higher loading of nanoparticulate catalysts, preformed particles comprising hundreds of atoms need to be intercalated. Given interlayer heights of less than 1 nm, this is, however, unlikely for kinetic reasons.

More recently, the synthetic clay sodium fluorohectorite, (NaHec, [Na_0.5_]^inter^[Mg_2.5_Li_0.5_]^oct^[Si_4_]^tet^O_10_F_2_) which belongs to a handful of layered compounds that show the long‐known^[14]^ but rare phenomenon of osmotic swelling, became available.^[15]^ Osmotic swelling is a thermodynamically allowed process^[16]^ and therefore produces liquid crystalline phases with a uniform separation of adjacent silicate layers. For NaHec nanosheets with 0.96 nm thickness and a median diameter of 20 μm,^[17]^ rotation of the nanosheets, even in very dilute suspensions (<1 vol %), is hindered and nematic liquid crystalline phases are formed instead of isotropic suspensions.^[18]^ As has been reported for titanate nanosheets,^[19]^ dilute aqueous dispersions of negatively charged NaHec nanosheets adopt a cofacial arrangement due to strong electrostatic repulsion. In this nematic state, adjacent Hec nanosheets are not only held in a coherent cofacial geometry, but are separated to long, well defined distances determined by the clay content, typically exceeding 50 nm. Loading these nematic phases with nanoparticles was previously proven by the intercalation of maghemite nanoparticles between the nanosheets.^[20]^

**Scheme 2:**
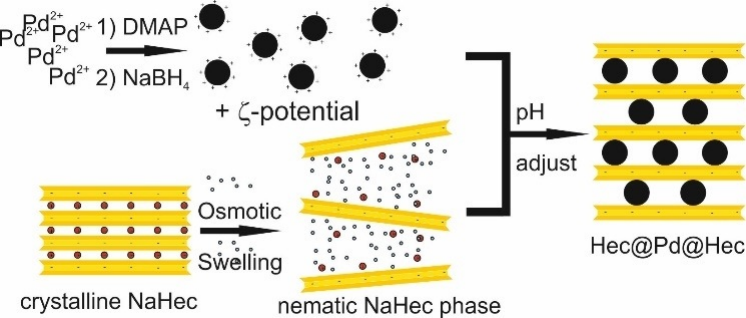
Schematic outline of the synthesis of Pd intercalated Hec (Hec@Pd@Hec).

As we will show here, this nematic nanosheet phase offers a scalable route to produce nanoparticulate catalysts between sub‐nanometer oxidic supports that resemble the model architectures (Scheme 1). Pdnanoparticles are first synthesized by established protocols^[21]^ and capped with 4‐dimethylaminopyridine (DMAP) yielding “nanoparticulate metal cations” that can easily diffuse into the open galleries between adjacent nanosheets similar to a cation exchange (Scheme 2). To probe the influence of the nanosheets on the properties of Pd nanoparticles, the mesostructured composite was tested in the oxidation of carbon monoxide (CO).

## Results and Discussion

### Synthesis and Characterization of Hec@Pd@Hec Catalysts

According to transmission electron microscope (TEM) images the as‐synthesized spherical nanoparticles had a narrow size distribution of 3.5±0.4 nm (Figure S1a). They were readily dispersible in water with a hydrodynamic diameter of 4.5±1.3 nm as determined by dynamic light scattering (DLS). The ζ‐potential could be tuned from +34 to +14 mV by adjusting the pH in the range from 6 to 12, respectively (Figure S1b). At the given and fixed cation exchange capacity, the surface charge density of the NaHec determines the number of nanoparticles required for charge balance and thus the loading can be tuned via the pH (Table 1).

**Table 1:** Weight fraction of Pd in the catalyst depending on the initial pH of hectorite and nanoparticle dispersion.

Sample	pH	ζ‐potential [mV]	Pd‐loading (ICP‐OES) [wt %]	Pd‐loading (SEM‐EDS) [wt %]
Hec@Pd65@Hec	9.5	28	65.2	67.8
Hec@Pd72@Hec	10.8	22	72.5	76.0

Typically, a 0.1 wt % dispersion of the Pd nanoparticles were added to a 1.5 wt % dispersion of a nematic phase of NaHec under vigorous stirring. At this NaHec content, the separation of adjacent parallel oriented nanosheets was found to be more than 60 nm by small angle X‐ray scattering in aqueous dispersion (Figure S2). This large gallery height and the positive surface potential of Pd nanoparticles allowed for fast incorporation (<30 seconds) of the catalyst whereupon hetero‐coagulation is triggered. Element mapping suggested a uniform loading of Pd (Figure S3). Moreover, as indicated by CHN analysis, the capping ligand could be completely removed by repeated centrifugation and washing (Table S1). To stress the sandwich confinement, we refer to the samples by Hec@Pdx@Hec where x corresponds to the weight fraction of Pd as determined by inductively coupled plasma optical emission spectroscopy (ICP‐OES). The loading was crosschecked by scanning electron microscopy with energy dispersive X‐ray spectroscopy (SEM‐EDS) (Table 1). Moreover, interlayer Na^+^ had been completely replaced according to ICP‐OES, SEM‐EDS (no signal at 1.04 keV, Figure S3) or X‐ray photoelectron spectroscopy (XPS, no signal for Na 1s at around 1070 eV, Figure S4) indicating that Na^+^ was completely replaced and that the negative charge of Hec nanosheets was fully balanced by the intercalated Pd nanoparticles.

As expected for such a quasi‐ion‐exchange, the weight fraction of Pd increased to a maximum of 72.5 wt % with decreasing surface potential of the nanoparticles. Contrary to the simple ion‐exchange route mentioned in the introduction, very high loadings were achieved by intercalation of positively charged nanoparticles. For instance, the sample containing 65.2 wt % Pd resulted in a stoichiometry of Pd_6.7_Mg_2.5_Li_0.5_Si_4_O_10_F_2_. For comparison, by simple ion exchange of Na^+^ for Pd^2+^ followed by reduction, the composition would be limited to Pd_0.25_Mg_2.5_Li_0.5_Si_4_O_10_F_2_.

**Figure 1:**
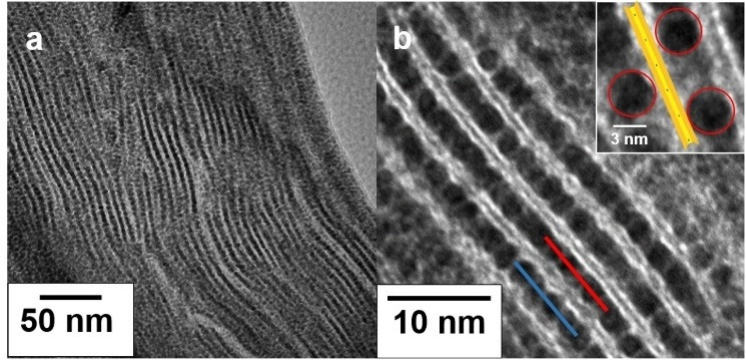
TEM images of cross sections of Hec@Pd65@Hec at different magnifications. The red and blue line were used for grey scale analysis (Figure S5). The inset shows adjacent Pd nanoparticles separated from each other.

Upon hetero‐coagulation, the nematic structure collapses to lamellar composites and adjacent Hec nanosheets sandwich the Pd nanoparticles (Figure 1). The nanoparticles are not densely packed, but separated from each other (Figure 1 b, inset). Each Pd nanoparticle is separated from the adjacent nanoparticle layer by exactly one silicate layer of 0.96 nm thickness. Since the Pd nanoparticle layers are randomly shifted relative to each other, thousands of architectures similar to what is sketched in Scheme 1 were obtained where a Pd nanoparticle is separated by a Hec nanosheet from an opposite mesopore (Figure 1 b, inset). This architecture was further confirmed by a grayscale analysis (Figure S5) of TEM images along a line of adjacent Pd nanoparticles in a layer (red and blue line in Figure 1 b). The nanoparticles retained their spherical shape after washing off the DMAP. In contrast, prolonged heating indeed caused some elongation of the nanoparticles (Figure S6).

**Figure 2:**
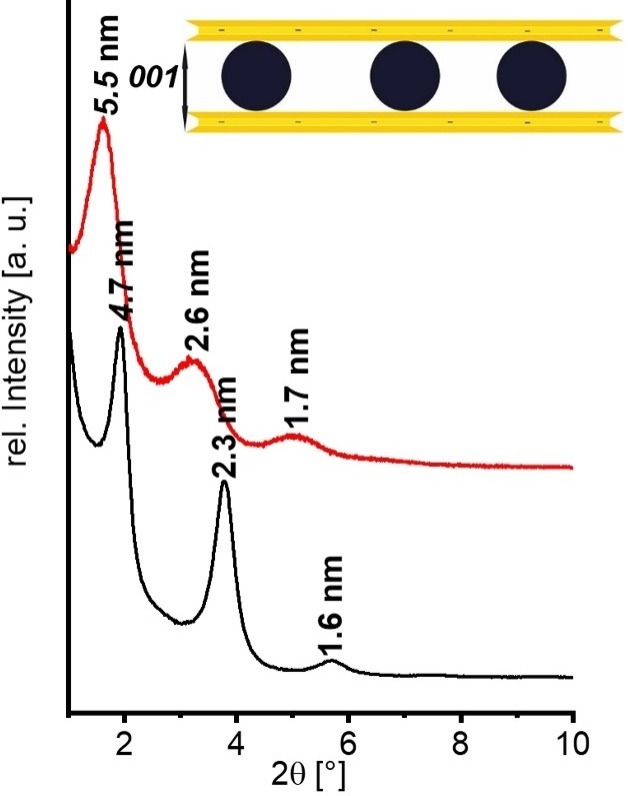
PXRD pattern of Hec@Pd65@Hec (black), and Hec@Pd72@Hec (red).

Since monomodal Pd nanoparticles were applied, the restacking upon hetero‐coagulation produces one‐dimensional periodic composite structures along the stacking direction. The periodicity was determined to be 4.6±0.7 nm by TEM analysis. At higher loadings (Hec@Pd72@Hec), few multilayers of Pd nanoparticles were formed in the interlayer space (Figure S7) which represent defects in the periodicity. Apparently, the surface charge density at pH 10.8 (22 mV ζ‐potential) was too low to accomplish charge balance of the anionic hectorite nanosheets purely in monolayers of cationic Pd nanoparticles. Powder X‐ray diffraction (PXRD) traces of textured samples confirm the one‐dimensional crystalline order. In good agreement with the TEM results for Hec@Pd65@Hec, a rational *00l* series with a periodicity of 4.7 nm was observed (Figure 2). Summing the thickness of a Hec nanosheet of 0.96 nm and the diameter of the nanoparticles of 3.5 nm a value of 4.46 nm would be expected. At a loading of 72 wt % (Hec@Pd72@Hec), the few defects of interstratified Pd double layers caused the *00l* series to be apparently shifted to 5.5 nm. Concomitantly, these defects lead to a greatly increased full width at half maximum suggesting that the observed shift was actually an artefact due to random interstratification of mono‐ and double‐layers. The X‐ray beam then averages between the different d‐spacings within its coherence length.

As already suggested by the TEM images, the Pd nanoparticles are not densely packed, but the Pd layers are porous as independently shown by Ar physisorption and CO chemisorption measurements (Table 2 and Figure S8). For instance, the d_50_ pore size for Hec@Pd65@Hec was 4.3 nm, which is in the same range as the size of the Pd nanoparticles, suggesting that some 50 % of the volume of the Pd nanoparticle layers is actually empty space. With randomly stacked layers the chances of having such a pore arranged opposite of a Pd nanoparticle (Scheme 1) are high.

**Table 2:** Results of Ar adsorption^[a]^ und chemisorption of CO.^[b]^

Sample	S_BET_ [m^2^ g^−1^]	Pore size [nm]	Pore volume [cc g^−1^]	Metal dispersion [%]
NaHec	4	/	/	/
Hec@Pd65@Hec	147	4.3	0.132	23.7
Hec@Pd72@Hec	87	3.8	0.088	19.9

[a] determined by Ar physisorption at 87 K. [b] determined by CO double isotherm method.

In contrast, the Ar‐isotherm of pristine NaHec revealed a nonporous structure with a BET surface as low as 4 m^2^ g^−1^. With this material, the galleries have collapsed and Ar has no access to the internal (interlayer) surfaces. The structure becomes porous only after intercalation of Pd nanoparticles acting as pillars.

The dispersion (ratio of surface to bulk atoms) for single, free‐floating, spherical Pd nanoparticles of 3.5 nm diameter is expected to be 32 %. Due to the good accessibility of the intercalated Pd nanoparticles, a surprisingly high experimental dispersion of 24 % for Hec@Pd65@Hec was measured. Apparently, only a small share of the surface became shielded by supporting it from two sides by Hec nanosheets.

### Evaluation of the Catalytic Activity

**Figure 3:**
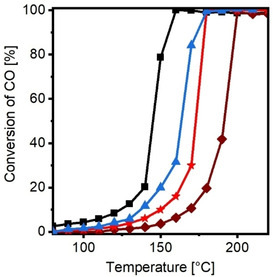
Light‐off curves for CO oxidation: Hec@Pd65@Hec (black), Hec@Pd72@Hec (blue), Pd_ext_@Hec (red), and Pd_ext_@Al_2_O_3_ (brown). Conditions: 50 mL min^−1^ (1 vol % CO, 1 vol % O_2_ balanced by N_2_).

The oxidation of carbon monoxide (CO) to carbon dioxide (CO_2_) was chosen as a simple test reaction to study the catalytic performance and to probe for an electronic‐metal‐support interaction due to the special architecture. This reaction is one of the most studied heterogeneous catalytic reactions due to its simplicity yet enormous importance for exhaust gas purification as CO is highly toxic.^[22]^ For each catalysis run the amount of catalyst was chosen to involve 1 mg of Pd in a feed gas stream of 50 mL min^−1^ (1 vol % CO, 1 vol % O_2_ in N_2_ carrier gas) and light‐off curves from 80 °C to 220 °C were recorded. Each catalyst was cycled three times. All three consecutive light‐off curves of Hec@Pd65@Hec are shown in Figure S9. In Figure 3 the third light‐off curve is presented (Figure 3 and Table 3).

**Table 3:** Catalytic light‐off behavior in CO oxidation.

Sample	T_10_ [°C]	T_50_[°C]	T_90_ [°C]	E_A_ [kJ mol^−1^]
Hec@Pd65@Hec	124	145	156	42
Hec@Pd72@Hec	138	163	173	51
Pd_ext_@Hec	150	172	177	49
Pd_ext_@Al_2_O_3_	169	191	198	57

For Hec@Pd65@Hec, which mimics the preferred architecture best (Scheme 1 and Figure 1), the temperature of 50 % conversion (T_50_) was found to be as low as 145 °C. The apparent activation energy *E_A_* determined at conversions below 10 % was 42 kJ mol^−1^ (Figure S10), which is lower than reported values for Pd metal supported on conventional supports such as γ‐Al_2_O_3_ or MgO (55–80 kJ mol^−1^)^[23]^ or silica (65–120 kJ mol^−1^).^[24]^

The good catalytic activity of Hec@Pd65@Hec might be related to several factors: First, influence of the structure inherent elements such fluoride. Second, stabilization of atomically dispersed Pd, third an influence of the mesoporous confinement, and fourth, an electronic interaction between support and metal as discussed in the introduction.

Although the structural fluoride is remote from the surface and not directly in contact with the Pd nanoparticles, we realize that such elements could have some influence on the catalytic behavior. This aspect can, however, only be addressed in future work when fluoride deficient layered supports will be investigated.

The Pd nanoparticles were dialyzed for several days before combining them with the nematic hectorite suspension. Dialysis is expected to remove smaller clusters. They still could have been freshly produced by dissolution during mixing with hectorite. Since the external basal planes of hectorite also carry a negative surface charge, they should be equally capable of stabilizing atomically dispersed Pd if indeed present. The same Pd nanoparticles (Pd_ext_@Hec, Figure S11a and Table S2) used to synthesize Hec@Pd65@Hec, but deposited on the outer surface of non‐swollen Hec crystals instead of being sandwiched in the interlayer space, showed a much higher T_50_ of 172 °C. This indicates that it is not, or at least not only, stabilization of smaller clusters or atomically dispersed Pd that improves the catalytic performance.

Hec@Pd65@Hec and Hec@Pd72@Hec loadings show similar pore size distributions while the metal dispersion of the latter is lowered by about 20 %. The few double layers of Pd nanoparticles observed in Hec@Pd72@Hec, however, had a huge detrimental effect on catalytic activity. T_50_ increased from 145 °C for Hec@Pd65@Hec to 163 °C for Hec@Pd72@Hec, which is already close to the value of only external Pd (Pd_ext_@Hec). The activation energy also increased (42 kJ mol^−1^, 51 kJ mol^−1^, 49 kJ mol^−1^ for Hec@Pd65@Hec, Hec@Pd72@Hec, and Pd_ext_@Hec, respectively). The reduction of the activity clearly is much larger than what is expected based on the smaller dispersion, which indicates that the mesoporosity may not be the determining effect for the good catalytic activity of Hec@Pd65@Hec.

It seems that the fourth factor, the special architecture might indeed be the determining factor for the activity. Applying a support with a less negative surface potential (−20 mV at a pH of 10) like γ‐Al_2_O_3_ loaded with 1 wt % Pd nanoparticles (Pd_ext_@Al_2_O_3_, Figure S11b and Table S2) yielded a catalyst with T_50_ of 191 °C and *E_A_* of 57 kJ mol^−1^ (Figure S10), values which are in good agreement with literature.^[23a]^ Although the chemistry of Al_2_O_3_ is different from Hec, this comparison suggests that the special support architecture potentially in collaboration with the fact that Pd has to counterbalance the large negative charge density (1 negative charge per 48 Å^2^ of support) of Hec might indeed have some influence.

**Figure 4:**
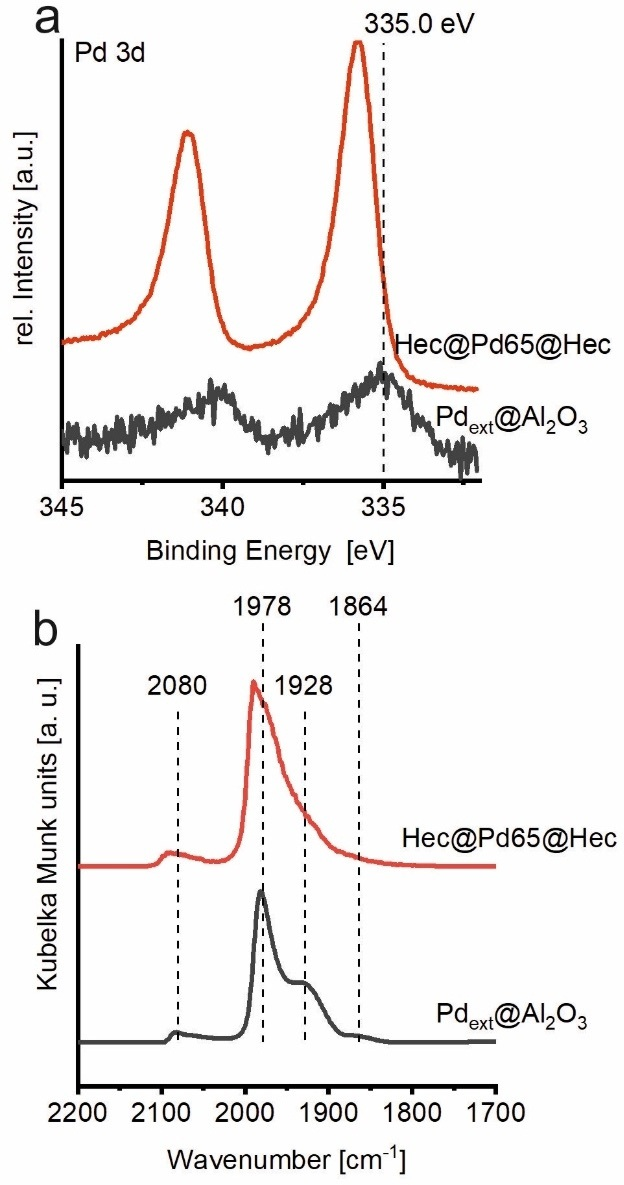
Characterisation of Pd nanoparticles of Hec@Pd65@Hec (red) and Pd_ext_@Al_2_O_3_ (black). a) XP spectra of Pd 3d region and b) DRIFT spectra of CO chemisorbed at 300 K to the surface of Pd at an equilibrium pressure of 2 mbar CO.

As mentioned earlier, Pd nanoparticles in Hec@Pd65@Hec have to balance the permanent negative charge of the Hec nanosheets. XPS of Pd 3d region of Hec@Pd65@Hec showed asymmetric signals of Pd 3d_5/2_ and Pd 3d_3/2_ at binding energies (BE) of 335.8 eV and 341.0 eV, respectively (Figure 4 a).

These values are shifted to higher energies as compared to bulk Pd metal at 335.0 eV^[25]^ or nanoparticles supported on Al_2_O_3_ (335.0–335.5 eV)^[26]^ or SiO_2_ (334.8–335.4 eV).^[27]^ This shift might be attributed to an electron deficient species Pd^δ+^.^[26a]^ Furthermore, as expected from the lower ζ‐potential of the Pd nanoparticles applied for Hec@Pd72@Hec the shift of the Pd 3d region was smaller (335.5 eV, Figure S12). Pd 3d_5/2_ of the same Pd nanoparticles applied in the synthesis of Hec@Pd@Hec, but supported on the external surface of NaHec (Pd_ext_@Hec) or γ‐Al_2_O_3_ (Pd_ext_@Al_2_O_3_) showed considerably lower BE of 335.3 eV and 335.2 eV, respectively (Figure 4 a and S12). These trends clearly show that both the negative charge of the nanosheets and the special architecture obtained by intercalation of Pd into the nematic Hec phase, indeed seem to have an influence on the electronic structure of Pd nanoparticles. This trend is also in line with the increasing performance for the oxidation of CO.

It has also been discussed that “cationic” Au species are important for higher catalytic activity in the case of Au catalysts.^[28]^ Furthermore, DFT calculations of Pd@zeolite FAU have indicated that positively charged Pd atoms lead to lower energy barriers assuming a Langmuir‐Hinshelwood mechanism.^[29]^ Based on DFT calculations, the higher activity of Pd^δ+^ species was attributed to weaker CO binding to positively charged Pd.^[23c]^

As the CO adsorption is very sensitive to the Pd surface constitution, the stretching vibration region of CO chemisorbed to the Pd surface of Hec@Pd65@Hec and Pd_ext_@Al_2_O_3_ was recorded by diffuse reflectance infrared Fourier transform spectroscopy (CO‐DRIFTS, Figure 4 b). Massive shifts of the stretching frequency were observed with increasing CO partial pressure up to 60 mbar CO due to dipolar coupling with increasing surface coverage (Figure S13). After outgassing of CO to an equilibrium pressure of 2 mbar CO, Pd_ext_@Al_2_O_3_ showed four bands centered at 2080, 1976, 1928, and 1864 cm^−1^. These can be attributed to different binding modes of CO to the surface of Pd, that are CO linearly bound to corners (2080 cm^−1^)^[30]^ and bridge bound CO on steps (1978 cm^−1^).^[31]^ The two broad bands at lower wavenumber (1928 and 1864 cm^−1^) are ascribed to bridge or three‐fold bonds on different planes.^[31]^ At the same equilibrium pressure of CO, the DRIFT spectrum of Hec@Pd65@Hec showed also four bands at 2094, 1991, 1945, and 1877 cm^−1^, all shifted to higher wavenumbers as compared to Pd_ext_@Al_2_O_3_ (Figure 4 b). A Pd surface with a partial positive charge as suggested for Hec@Pd65@Hec can back‐donate less electrons to the antibonding CO 2π* orbital that results in a stronger C−O bond and a wavenumber shift to higher wavenumbers.^[31a]^ A weaker back‐donation would also lead to weaker adsorption of the CO molecules to the Pd surface.

**Figure 5:**
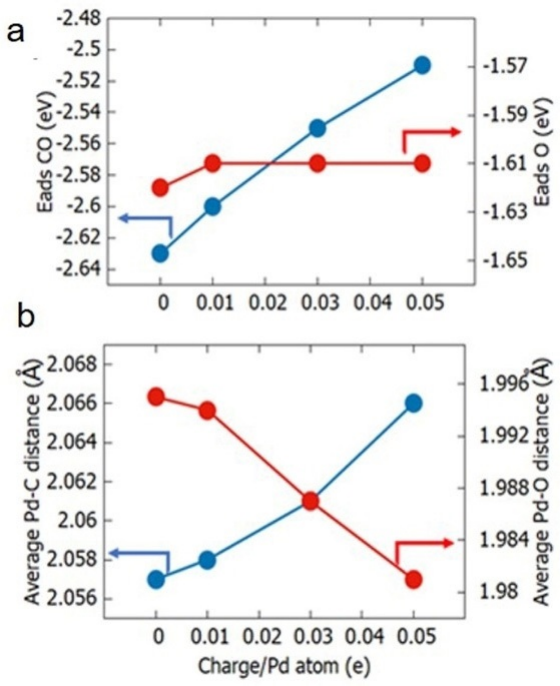
a) Calculated adsorption energies of low coverage CO (blue) and O (red) as a function of nanoparticle charge. b) Average Pd−C (blue) and Pd−O (red) bond lengths as a function of nanoparticle charge.

In order to further corroborate the hypothesis of the influence of a positive charge of Pd nanoparticles on the adsorption strength on CO, DFT calculations were performed to examine the adsorption of CO/O upon a representative nanoparticle model: icosahedral Pd_147._ This nanoparticle has a diameter of 1.5 nm and contains (*111*) microfacets which closely approximate the extended (*111*) surface. CO and atomic oxygen were adsorbed onto the hcp hollow sites of the metallic particle at a local microfacet coverage of *θ*=0.1 (details of the model (Figure S14–S17) and methods are provided in the supporting information). Adsorption energies for CO were observed to decrease linearly with increasing positive charge on the particle over the considered range (of Pd_147_ to Pd_147_^7+^), while the O adsorption energies were unchanged (Figure 5). Concomitant with the reduction in CO adsorption energy, was a lengthening of the average Pd−C bond from 2.057 Å to 2.066 Å, and a shortening of the C−O bond, from 1.197 Å to 1.187 Å. Therefore, the present DFT calculations are consistent with the experimental DRIFTS results. This finding is understood in terms of molecule‐metal bonding models that suggest the depletion of electrons in the metal d states near the Fermi energy reduce the occupation of the net‐bonding π channel between CO and Pd. This in turn weakens adsorption and strengthens the internal C−O bond.^[32]^ The nanoparticle charge may thus enhance CO oxidation by reducing CO poisoning. Furthermore, computational data^[29]^ revealed lower energy barriers in the catalytic cycle for positively charged Pd sites due to the altered binding strength of CO that is in line with the observed lower activation energy of Hec@Pd@Hec.

**Figure 6:**
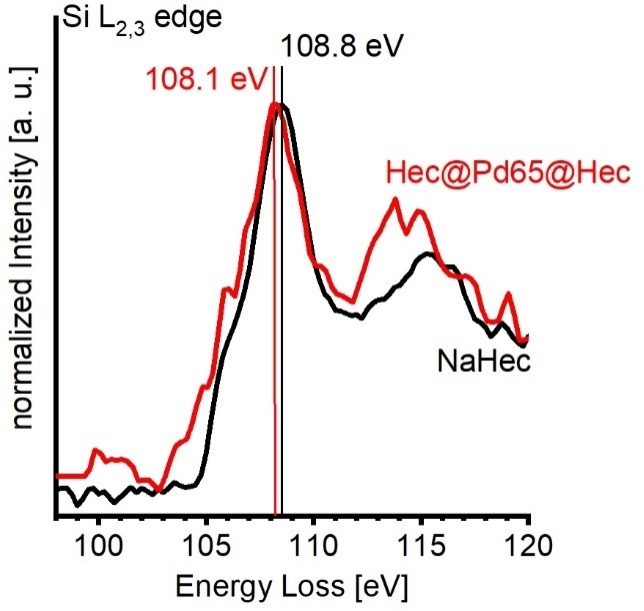
ELL spectra at the Si L_2,3_ edge of NaHec (black) and Hec@Pd65@Hec (red).

For Hec@Pd@Hec, the high permanent negative charge density inherent to this support might not be the only source of hole formation in intercalated Pd. It has long been shown that for the special architecture (Scheme 1, Figure 1), charge transfer between metal and ultrathin yet neutral oxide layers may occur and thus have an influence on the work function of the metal.[^5^, ^6^] It was found that an ultrathin layer of SiO_2_ deposited on Mo(*112*) increased the metal work function by 0.5–1 eV due to dipole effects arising from charge transfer from the metal to the oxide.^[6b]^ To probe such a possible electronic interaction between the hectorite nanosheets and the Pd nanoparticles leading to an additional charge transfer from Pd to silicate support, electron energy loss spectra (EELS) at the Si L_2,3_ edge were measured (Figure 6).

It revealed a chemical shift of both the white line at about 109 eV and the resonance at around 116 eV to lower energy losses for Hec@Pd65@Hec as compared to pristine NaHec. A chemical shift to lower energy losses of about 0.8 eV was also detected at the Si K edge (Figure S18). EELS thus suggests a slight but significant reduction to a Si^x^ (*x*< +4) species upon intercalation of Pd nanoparticles corroborating an electronic interaction between Pd and the support.^[33]^ As has been observed for model systems[^5^, ^6^] and although the Pd already carries a positive charge for reasons of charge neutrality, “coating” the Pd nanoparticles triggers an additional transfer of electron density to Si in the hectorite structure. This also resembles observations by *Li* et al.^[34]^ who synthesized Pd nanocubes covered with the Cu‐containing MOF HKUST‐1. They also reported similar shifts of Pd 3d BE as determined by XPS to higher energies, while concomitantly the Cu 2p BE is lowered. This was attributed to Cu‐O groups acting as electron acceptors.^[32a]^

## Conclusion

For model architectures of ultrathin layers of oxides deposited on noble metals modulations of the metal electronic structures have long been established and are advantageous for catalytic activity. These architectures can be mimicked at bulk scale by sandwiching positively charged metal nanoparticles between negatively charged clay nanosheets. Like for the model system, a charge transfer from the Pd nanoparticles to the nanosheets was observed by XPS, EELS, and CO‐DRIFTS and a higher catalytic activity in CO oxidation was observed as compared to Pd on conventional supports such as γ‐Al_2_O_3_. The synthesis route via intercalation into nematic phases of anionic nanosheets is certainly not restricted to Pd metals nor to hectorite nanosheets, but is applicable for a broad spectrum of metal nanoparticles of various sizes and shapes^[35]^ on one side, and other liquid crystalline supports like lepidocrocite‐type titanates,^[15b]^ or layered antimony phosphates[^15c^, ^15e^] on the other side. Needless to say, the concept can also be extended to catalytically more attractive alloy nanoparticles.^[36]^

## Conflict of interest

The authors declare no conflict of interest.

## Supporting information

As a service to our authors and readers, this journal provides supporting information supplied by the authors. Such materials are peer reviewed and may be re‐organized for online delivery, but are not copy‐edited or typeset. Technical support issues arising from supporting information (other than missing files) should be addressed to the authors.

Supplementary
