# Supplementary material for: Nanoparticles Supported on Sub‐Nanometer Oxide Films: Scaling Model Systems to Bulk Materials
Source: Angew Chem Int Ed Engl. 2021 Jan 28;60(11):5890–7. doi: 10.1002/anie.202015138 (PMC7986867; doi:10.1002/anie.202015138)
Supplement: Supplementary file 1 — Supplementary [file ANIE-60-5890-s001.pdf]

Angewandte Chemie
Eine Zeitschrift der Gesellschaft Deutscher Chemiker

Supporting Information

Nanoparticles Supported on Sub-Nanometer Oxide Films: Scaling Model Systems to Bulk Materials

*Kevin Ament, Nicolas Köwitsch, Dianwei Hou, Thomas Götsch, Jutta Kröhnert, Christopher J. Heard, Annette Trunschke, Thomas Lunkenbein, Marc Armbrüster, and Josef Breu**

anie_202015138_sm_miscellaneous_information.pdf

Table of Contents

1 Experimental Procedures

2 Results and Discussions

2.1 Characterization of Pd nanoparticles

2.2 PXRD of crystalline and nematic NaHec.

2.3 Elemental analysis of Hec@Pd65@Hec

2.4 XPS survey spectrum of Hec@Pd65@Hec

2.5 Grayscale analysis of Pd layers in Hec@Pd65@Hec

2.6 TE micrograph after prolonged heating

2.7 Cross section of Hec@Pd72@Hec

2.8 Physisorption isotherms

2.9 Cycling test of Hec@Pd65@Hec

2.10 Arrhenius plots

2.11 Characterisation of reference catalysts

2.12 XP spectra of Pd 3d region of reference materials

2.13 Isotherms of CO chemisorbed to the Pd surface

2.14 Additional computational information

2.15 EEL spectroscopy at Si K edge

3 Author Contributions

4 References

1 Experimental Procedures

Materials

$PdCl_2$ (99.999% Pd, Premion), NaCl, 4-Dimethylaminopyridine (DMAP), NaOH and $NaBH_4$ were purchased from Alfa Aesar. γ-Al_2O_3 with high surface area was purchased from Alfa Aesar. The pellets were grounded to a powder before use. The used water was of MilliQ quality (18.2 MΩ).

Catalyst synthesis

Synthesis of Pd nanoparticles:
The synthesis of Pd nanoparticles was executed using a modified literature procedure.[1] Palladium(II) chloride (235 mg, 1.33 mmol) and Sodium chloride (155 mg, 2.66 mmol) were dissolved in 40 ml water and 4-Dimethylaminopyridine DMAP (833 mg, 6.82 mmol) in 80 mL water was added. After 20 min of stirring $NaBH_4$ (110 mg, 2.91 mmol) in 11 ml water was added dropwise under vigorous stirring resulting in a black dispersion. After 2 h the nanoparticle dispersion was dialyzed in 4 l water with water changes after 12 and 24 hours.

NaHec was synthesized via melt synthesis and is described elsewhere.[2]

Synthesis of Hec@Pd@Hec:
For the synthesis of the catalysts the hectorite was delaminated to a 1.5 wt% dispersion to achieve a layer distance of about 60 nm. Both the aqueous particle and hectorite dispersions were adjusted with NaOH resulting in surface potentials of the nanoparticles of 28 mV or 22 mV, respectively.The nematic hectorite suspension was added rapidly to the nanoparticle dispersion under vigorous stirring. Visible flocculation appeared within 30 seconds. The black flocculate was separated from the supernatant by centrifugation. DMAP was removed by repeated washing cycles. Finally, the catalysts were freeze dried to obtain a fluffy powder.

Synthesis of $Pd_{ext}Hec$:
To a dispersion of Pd nanoparticles (1 mg/ml) crystalline NaHec was added under stirring. After 24 h the resulting black solid was separated by centrifugation and washed several times. The catalyst was freeze dried to obtain a fluffy powder.

Synthesis of $Pd_{ext}Al_2O_3$:
To a dispersion of Pd nanoparticles (1 mg/ml) was added γ-Al_2O_3 under stirring. The solvent was slowly removed under stirring at 80 °C. The catalyst was finally dried at 130 °C.

Measurement and Characterization Techniques
Dynamic light scattering (DLS) and determination of ζ-potential were recorded on a Litesizer 500 (Anton-Paar).
CHN analysis was acquired with an Elementar Vario EL III.

Powder X-ray diffraction (PXRD) measurements were done using a STOE Stadi P diffractometer. $Cu_{K\alpha 1}$ radiation and a Mythen 1K silicon strip-detector were used. PXRD of traces of textured samples were recorded on a Bragg-Brentano type diffractometer (Empyrean, PANalytical) with nickel filter and $Cu_{K\alpha}$ radiation (λ = 1.54187 Å).

SAXS data were measured using a “Double Ganesha AIR” system (SAXSLAB,Denmark). The X-ray source of this laboratory-based system is a rotating anode (copper, MicroMax 007HF, Rigaku Corporation,Japan) providing a microfocused beam. The data was recorded by a position sensitive detector (PILATUS 300 K, Dectris)

Transmission electron microscopy (TEM) images were acquired using a JEOL JEM-2200FS (200 kV). For cross sectional TEM the powder was embedded into a resin (EPO-TEK 301) and was cut with an Ar beam into thin slices using a Jeol Cryo Ion Slicer.
Scanning electron microscopy (SEM) images and energy dispersive spectroscopy (EDS) were recorded on a FEI Quanta FEG 250.

Photoelectron spectroscopy (XPS) was conducted on a PHI 5000 Versa Probe III fitted with an Al Kα excitation source and spectra were analyzed with Multipak software pack. Spectra were referenced to C 1s at 284.8 eV.
Electron energy loss spectroscopy (EELS) measurements were conducted using a double-corrected JEOL JEM-ARM200F microscope, operated at 200 kV and equipped with a Gatan GIF Quantum imaging filter with DualEELS capabilities. Plural scattering was removed from all spectra by Fourier ratio deconvolution.

Adsorption isotherms were recorded on a Quantachrome Autosorb-1 with Ar as adsorbate at 87 K. The isotherms were evaluated using Brunauer-Emmet-Teller (BET) method and pore size distribution was evaluated with NLDFT. Metal surface was acquired with a Quantachrome Autosorb-1 with CO at 35 °C using the double isotherm method.

To determine the elemental composition, to about 20 mg of the sample was added a mixture of 1.5 mL 30 wt% HCl (Merck), 0.5 mL of 85 wt% H_3PO_4 (Merck), 0.5 mL 65 wt% HNO_3 (Merck) and 1 mL of 48 wt% HBF_4 (Merck). The sample was digested in a MLS 1200 Mega microwave digestion apparatus for 6.5 min and heated at 600W (MLS GmbH). The sample was allowed to cool to room temperature and the clear solution was diluted to 100 mL and analyzed with a Varian Vista-Pro radial ICP-OES.

Diffuse reflectance infrared Fourier transform (DRIFT) spectra were collected on a Cary 680 FTIR spectrometer from Agilent equipped with a MCT detector and a Praying Mantis™ low temperature reaction cell from Harrick. Spectra were recorded at a spectral resolution of 2 cm^{-1} and accumulation of 1024 scans. Samples were dried at 130 °C under a flow of Ar (50 ml/min) over night and then reduced at 150 °C under a flow of H_2 (10 vol%) for 1 h. To remove H_2 the samples were evacuated at 150 °C for 30 min and then let allow to cool to room temperature. Spectra were taken at 300 K. CO isotherms were recorded by dosing CO at increasing equilibrium pressures ranging from 0.03 to 60 mbar. The spectra are presented in Kubelka–Munk units $F(R_\infty) = (1 - R_\infty)2/2R_\infty$

Catalysis
Catalytic tests were conducted in a fixed bed micro reactor with an internal diameter of 4 mm. The desired amount of catalyst was mixed with quartz to achieve an overall loading of 500 mg. The reactor was heated using a circular kiln. To record light-off curves the temperature was raised in 10 °C steps and the temperature was held for 12 min before analysis. A reactant mixture of 1 vol% CO, 1 vol% O_2 und 98 vol% nitrogen with a constant flow of 50 ml/min under atmospheric pressure was injected into the reactor. The composition of the gas mixture leaving the reactor was monitored using an Agilent 6890N gas chromatograph equipped with a 30 m GS CARBONPLOT column and a thermal conductivity detector. Prior to catalysis, the catalyst was conditioned for 2 h in the reactant mixture stream at 200 °C for 2 h.

Computational Models and Methods
Method
Geometry optimisation and calculation of activation energies for CO oxidation were performed using the periodic density functional method within the VASP 5.4 package,[3] employing the projector augmented-wave method,[4] and the Perdew-Burke-Ernzerhof exchange-correlation functional[5] with Grimme’s D3 dispersion correction[6] and BJ damping.[7] The wavefunction expansion was truncated at 400 eV, and the Brillouin zone was approximated by the Gamma point only (except in slab calculations, for which a 4x4x1 Monckhorst-Pack[8] k point grid was employed). Electronic and force convergence criteria for local geometry optimization were set to 1x10-6 eV and 2x10-2 eV/Å, respectively. Gaussian smearing was included, with a smearing width of 0.1 eV. All calculations were performed spin-unpolarized, except for the case of molecular oxygen, which exhibits a triplet ground state. Dipolar corrections to the energy were included in all three dimensions. The electrostatic potential was verified to converge to a stable vacuum level for each charged particle system, in order to ensure spurious electrostatic effects between images were avoided.

SUPPORTING INFORMATION

To model the palladium nanoparticles in experiment, unsupported icosahedral palladium nanoparticles comprising 147 atoms (approx. 1.5 nm diameter) were generated within a cubic box of 25x25x25 Å. The structure of the particle was locally optimised for each charge state, from initial structures for which the surface Pd-Pd distances were approximately 2.75 Å; in-line with the extended (111) surface nearest-neighbour distance. This nanoparticle model includes (111) microfacets, and contains all important CO adsorption sites (top, bridge, fcc hollow and hcp hollow) that are appropriate for a pristine (111) surface or larger nanoparticle. The model therefore approximates the effects of surface undercoordination and curvature appropriate for the experimental 3.5 nm particle. The charged nanoparticles were generated via explicit removal of electrons corresponding to a per-Pd charge of +0.01e-, +0.03e- and +0.05e- (one, four and seven electrons removed, respectively). Adsorption was modelled by the addition of a single adsorbate to the pristine nanoparticle, to the hcp hollow site closest to the centre of a (111) microfacet. Adsorption energies of adsorbates CO (Eads(CO)) and atomic O (Eads(O)) were calculated according to the following equations,

Eads(CO) = E(CO@Pd147) – E(Pd147) – E(CO)(g)

Eads(O) = E(O@Pd147) – E(Pd147) – 0.5 E(O2)(g)

where the isolated Pd147 particle and CO (rC-O = 1.14 Å) and O2 (rO-O =1.23 Å) molecules are calculated in the same cubic cell. The molecular oxygen reference was calculated with spin polarization, to account for the triplet oxygen ground state.

2 Results and Discussion

2.1 Characterization of Pd nanoparticles

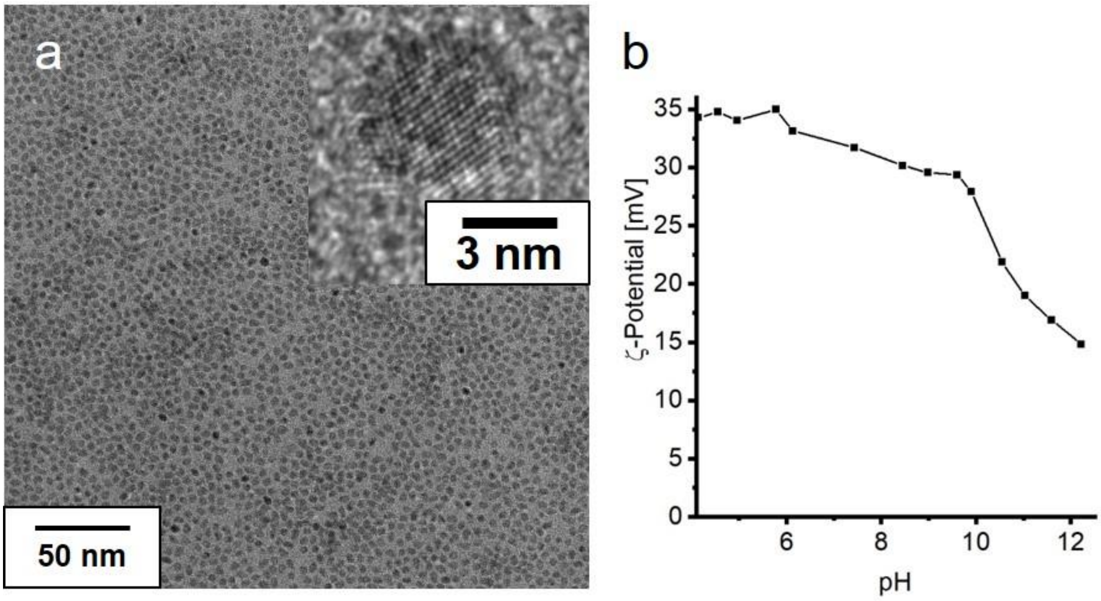

Figure S1. a) TEM image of monodisperse Pd nanoparticles. Inset: High magnification image. b) ζ-Potential versus pH for DMAP stabilized Pd nanoparticles in 0.1M NaCl solution. Below a pH of 5 the nanoparticles aggregated quickly due to the protonation of the amino function of DMAP.

2.2 SAXS of nematic NaHec

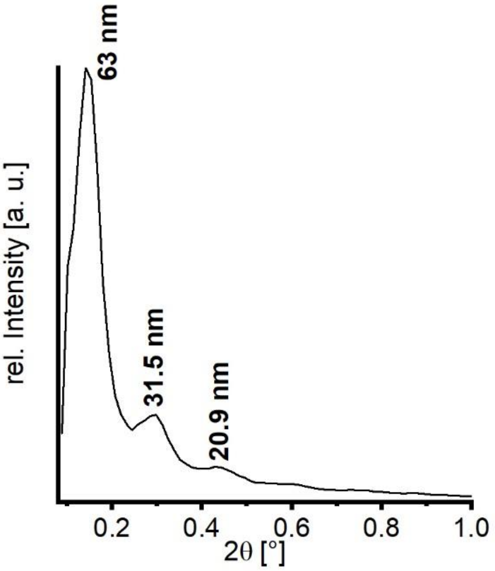

Figure S2. SAXS of a 1.5 wt% swollen nematic NaHec phase.

2.3 Elemental analysis of Hec@Pd65@Hec

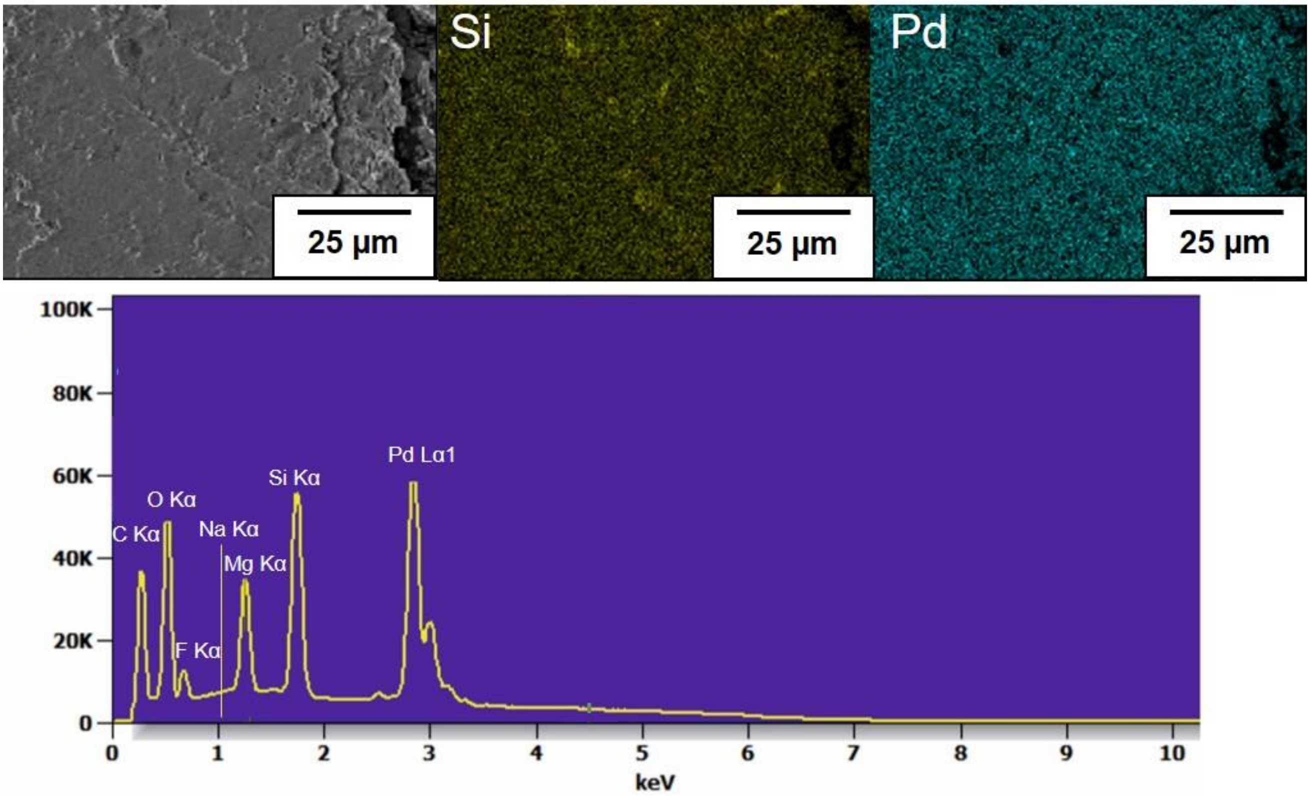

Figure S3. SEM-EDS elemental mapping of Hec@Pd65@Hec.

Table S1: Results of CHN analysis after washing.

Sample	Fraction of C [wt%]	Fraction of H [wt%]	Fraction of N [wt%]
Hec@Pd65@Hec	0.03	0.0	0.00
Pd@Hec72@Hec	0.04	0.0	0.02

2.4 XPS survey spectrum of Hec@Pd65@Hec.

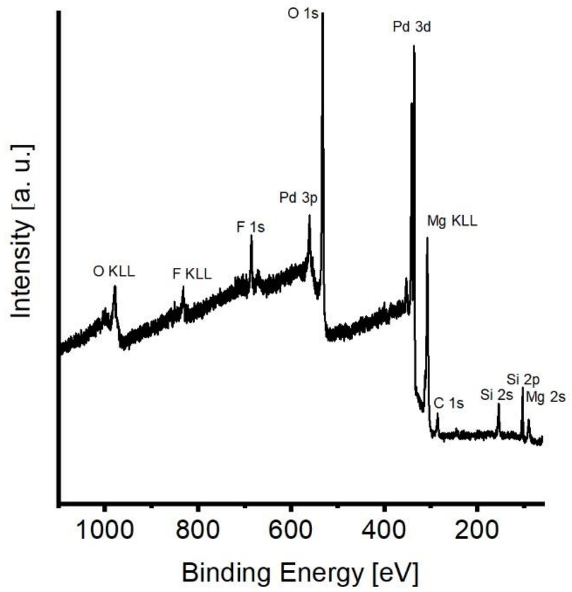

Figure S4. XPS survey spectrum of Hec@Pd65@Hec.

2.5 Grayscale analysis of Pd layers in Hec@Pd65@Hec

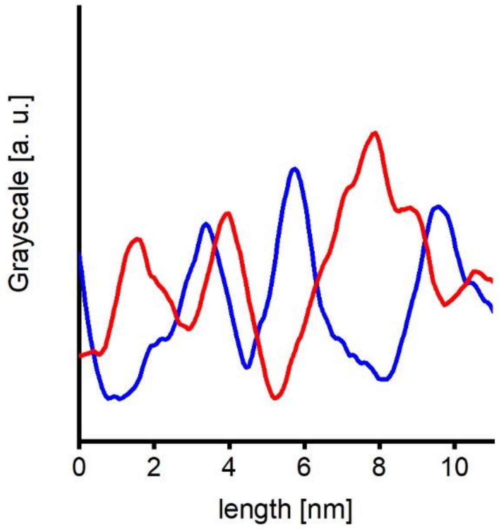

Figure S5. Grayscale analysis of two adjacent monolayers of Hec@Pd65@Hec separated by one nanosheet. It can be observed that nanoparticles of adjacent layers are shifted against each other.

2.6 TE micrograph after prolonged heating

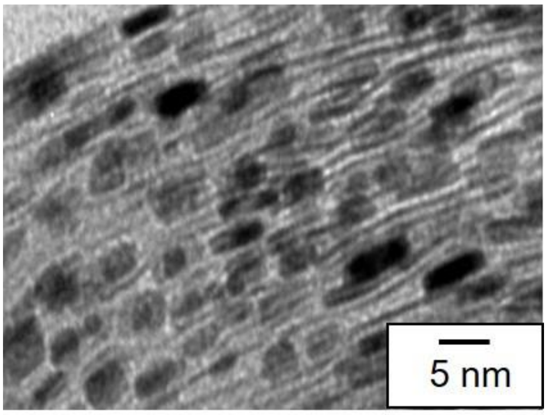

Figure S6. TE micrograph of Hec@Pd65@Hec after heating to 700 °C in Ar atmosphere for 10 h.

2.7 Cross section of Hec@Pd72@Hec

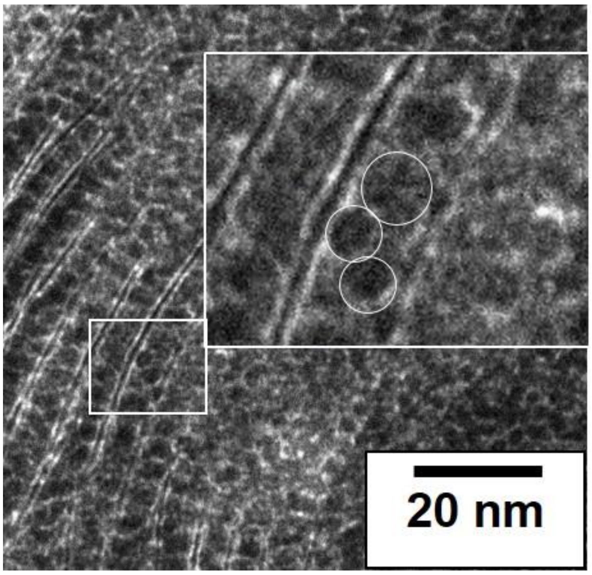

Figure S7. TE micrograph of cross section of Hec@Pd72@Hec showing domains with double layers of nanoparticles between the nanosheets.

2.8 Physisorption isotherms

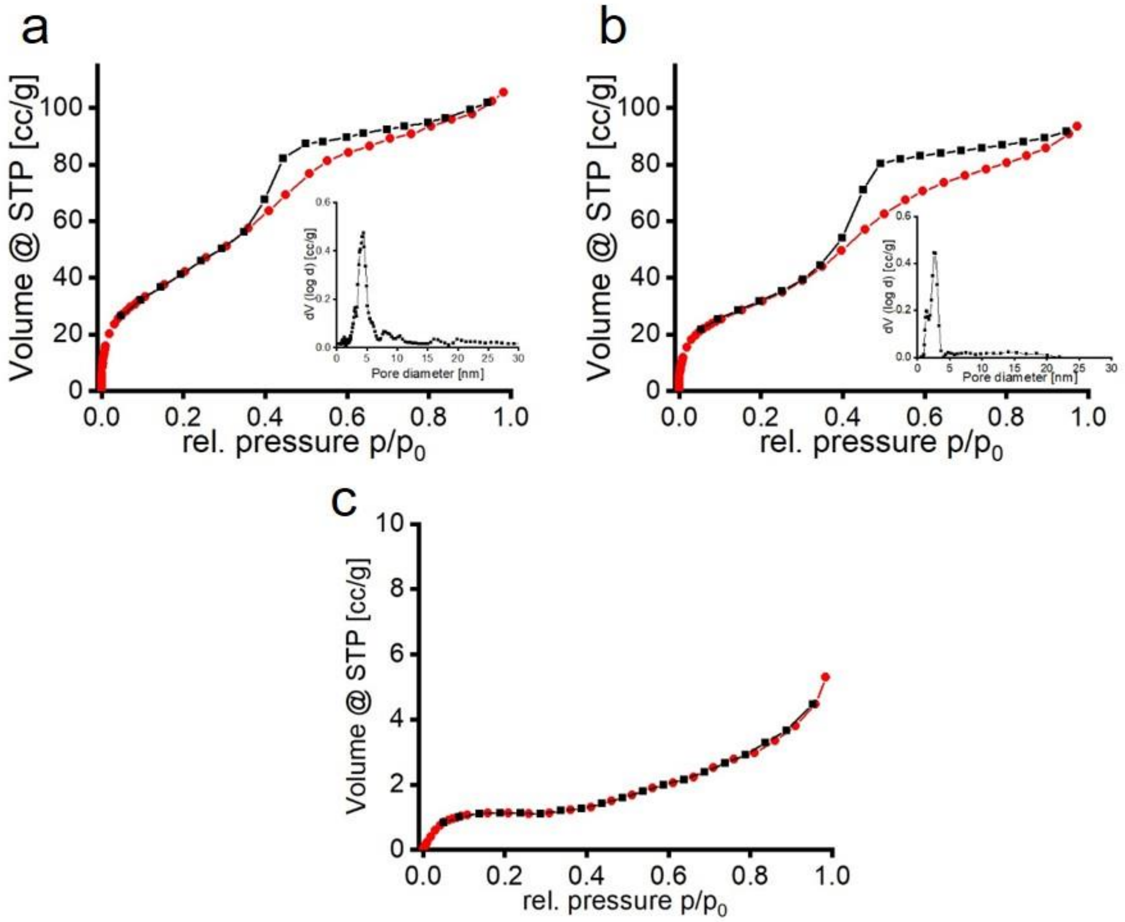

Figure S8. Ar-Isotherms and pore size distribution of a) Hec@Pd65@Hec, b) Hec@Pd72@Hec, and c) pristine NaHec. Hec@Pd@Hec samples show type IV(a) isotherms that correspond to a mesoporous network. The shape of the hysteresis can be attributed to the H2(b) type. This type of hysteresis in the desorption branch normally occurs due to pore necks of a larger size distribution. The distance of the particles can be seen as necks between the pores. As the particles have a size distribution, different sized necks are comprehensible.[9]

2.9 Cycling test of Hec@Pd65@Hec

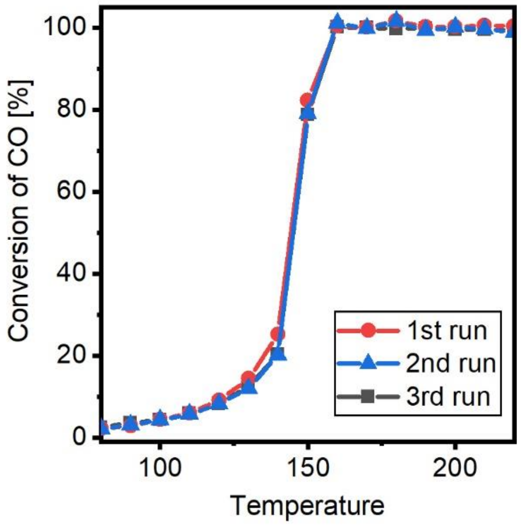

Figure S9. Cycling test of three runs of Hec@Pd65@Hec.

2.10 Arrhenius plots

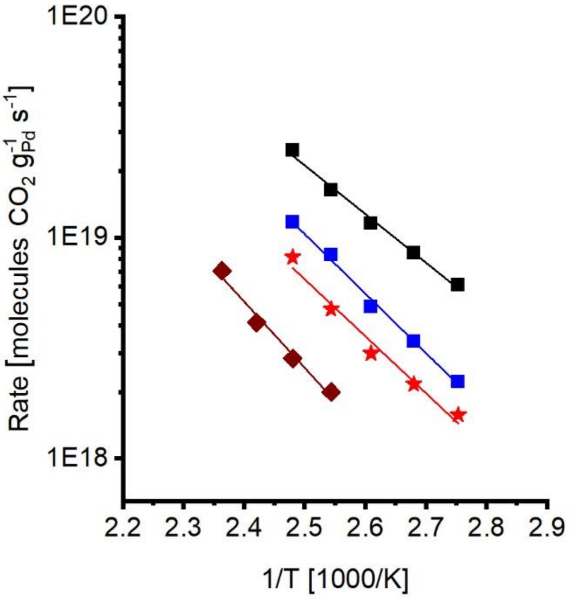

Figure S10. Arrhenius plot of Hec@Pd65@Hec (black), Hec@Pd72@Hec (blue), Pd_{ext}@Hec (red), and Pd_{ext}@Al_2O_3 (brown).

2.11 Characterization of reference catalysts

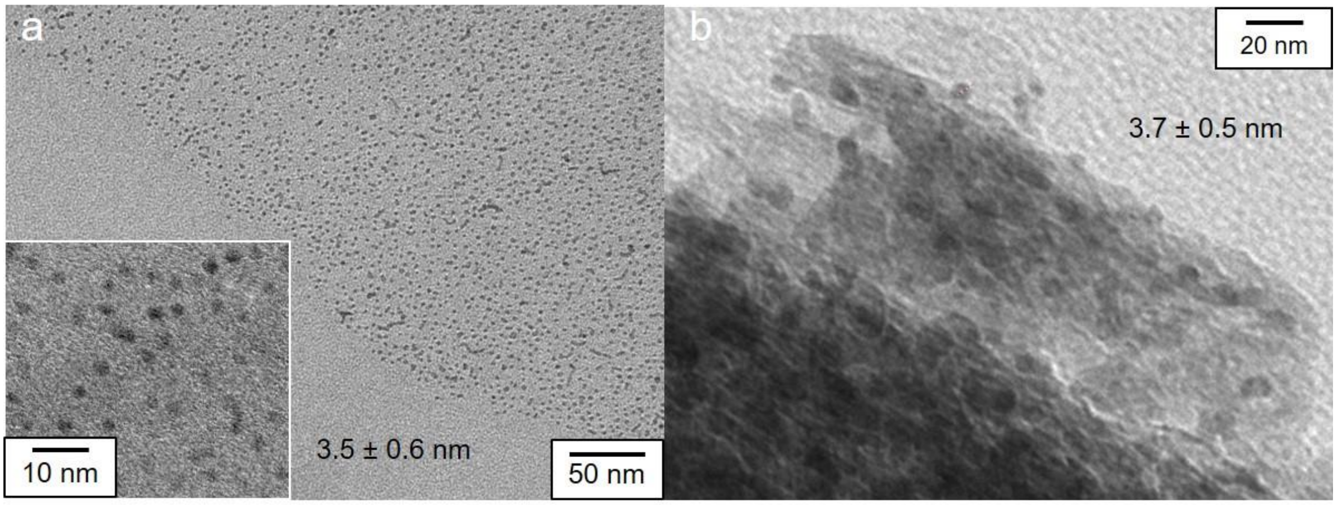

Figure S11. TE micrographs of a) Pd_{ext}@Hec and b) $Pd_{ext}@Al_2O_3$

Table S2. Characterization of reference materials.

Sample	Loading [wt%][a]	S_{BET} [m^2/g][b]	Pore size [nm][b]	Average Pd size [nm][c]	Metal dispersion [%][d]
$Pd_{ext}@Al_2O_3$	0.97	156	7.6	3.7 ± 0.5	22.7
Pd_{ext}@Hec	0.98	4.7	/	3.5 ± 0.6	19.6

[a] determined by ICP-OES, [b] determined by Ar physisorption at 87 K, [c] core sized determined by TEM, [d] determined by CO double isotherm method.

2.12 XP spectra of Pd 3d region of reference materials

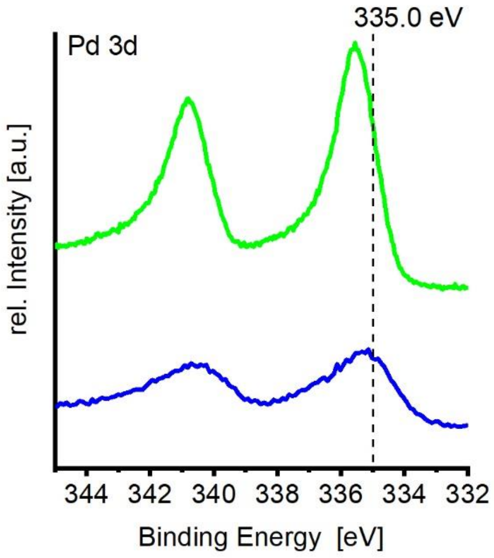

Figure S12. XP spectra of Pd 3d region of Pd_{ext}@Hec (blue) and Hec@Pd72@Hec (green).

2.13 Isotherms of CO chemisorbed to the Pd surface

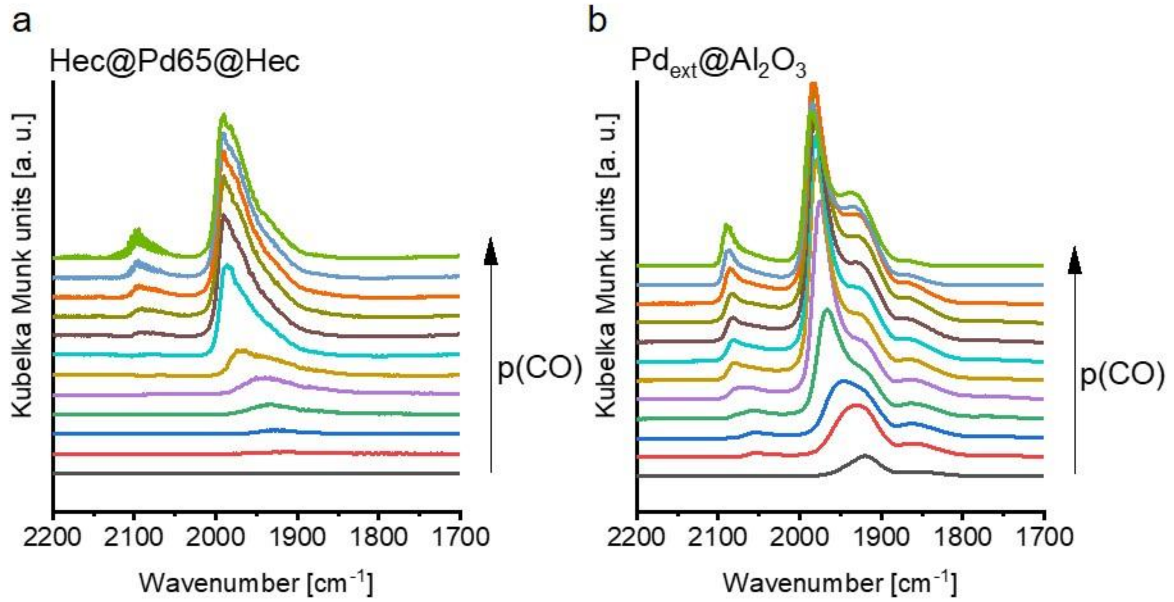

Figure S13. DRIFT spectra of CO-stretching vibration recorded at increasing CO equilibrium pressure of a) Hec@Pd65@Hec and b) Pd_{ext}@Al_2O_3.

2.14 Additional computational information

Electronic Density of States

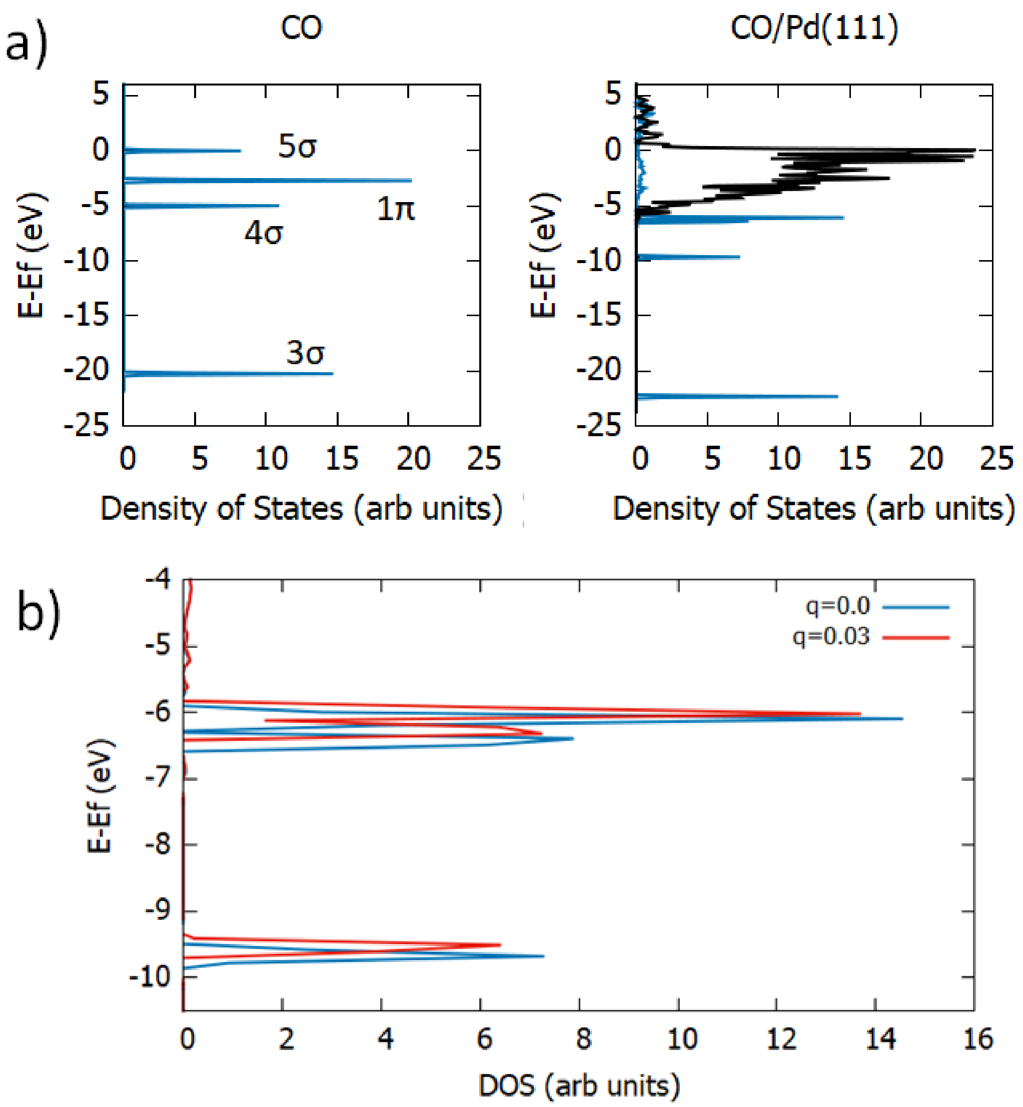

Figure S14. a) Projected density of states for an isolated CO molecule and CO upon adsorption to neutral Pd hcp site at low coverage. CO s/p states have blue colour Pd d states have black colour. b) CO s/p states upon adsorption to neutral and charged Pd (q=0.03e/atom).

The electronic structure of isolated and adsorbed CO is calculated within VASP, by projection of the electronic density of states onto atomic orbitals and separated into single atom contributions. The free CO molecule shows the hybridization of atomic orbitals into σ and π molecular orbital (MO) channels. Adsorption to the Pd surface induces an energetic downshift of adsorbate states and hybridization of the orbitals with the Pd s/d band. Figure S9a shows the full s/p states of CO and the d orbitals of Pd. Increasing Pd electron depletion leads to a relative upshift in CO adsorbate electronic states with respect to the Fermi energy. Figure S9b shows the upshift of states originating from CO 4σ and 1π orbitals.

Surface Calculations

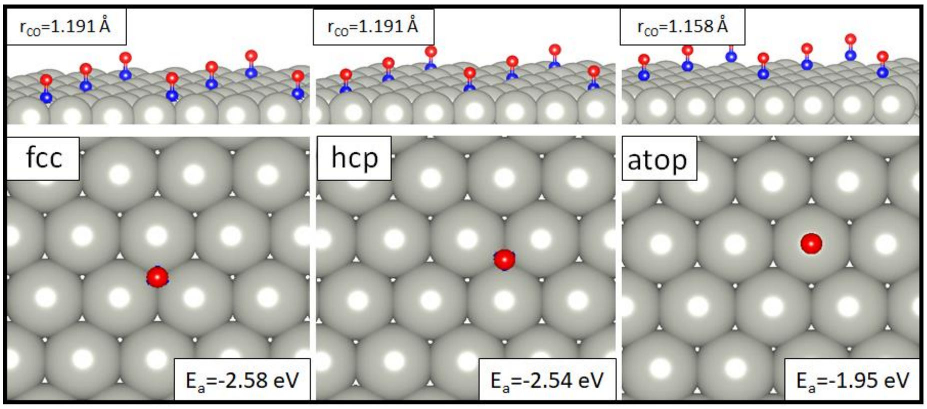

Figure S15. Adsorption energies of CO on the Pd(111) surface in fcc, hcp and top sites at a coverage of 1/9.

In order to identify the effect of undercoordination and cluster curvature on the adsorption properties on Pd, we calculated the adsorption of CO upon a pristine (111) surface. On the surface, the CO molecule was found to prefer hollow sites to top sites, while bridge sites were not found to be stable with respect to barrierless migration to a hollow site. Therefore, upon the nanoparticle only hollow sites were considered, with hcp hollows always found to be preferred over fcc sites. The adsorption is found to be moderately enhanced upon the nanoparticle, by less than 0.1 eV. Hence the 1.5 nm nanoparticle is a good approximation to the pristine extended surface, and therefore for the 3.5 nm nanoparticle in experiment.

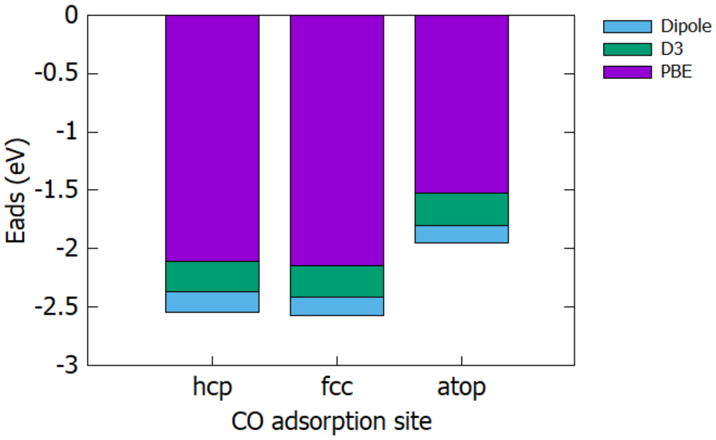

Figure S16. Energetic contributions to CO adsorption energy upon Pd(111). The dipole and D3 dispersion corrections provide a constant stabilisation for hcp, fcc and top adsorption modes.

The various contributions to adsorption energy are separated for a model of low-coverage CO adsorption on the pristine, neutral Pd(111) surface (θ = 1/9). Dispersion and dipolar corrections are found to provide a near-constant addition to adsorption energy of -0.27 eV and -0.16 eV, respectively, irrespective of adsorption mode. Neglect of these contributions gives GGA-only adsorption energies of -2.14 eV, -2.11 eV and -1.52 eV in fcc, hcp and top sites, respectively: in accord with literature values at the same level of theory.[10]

Coverage Effects

The role of particle charge in affecting the lateral repulsion between adsorbates was investigated. The coverage dependence of CO and O adsorption was modelled by occupation of a single (111) microfacet of Pd147 by adsorbates, in the range θ = 0.1 - 0.6 (between one and six adsorbates upon one microfacet). It is known that the preferred adsorption mode for CO varies with coverage. However, as the aim is to identify the effect of charge on energetic trends, rather than to locate the optimal adsorbate surface structure, hcp hollow sites were chosen as occupation sites for both species at all coverages. Increasing particle charge weakens the adsorption for all coverages of CO, with a constant upward shift in Eads. For O adsorption, the destabilisation of O on the surface is a stronger function of coverage than CO. Additionally, the trend as a function of charge is different between CO and O. While CO is destabilised in a constant manner, oxygen is found to exhibit a charge effect, in which higher excess positive charge leads to a stronger destabilisation.

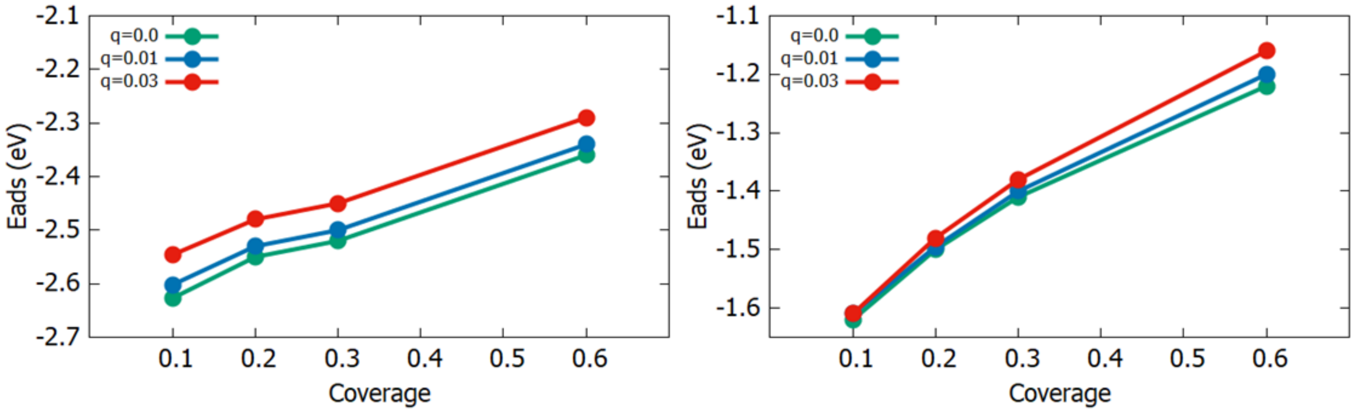

Figure S17. Coverage dependence of CO (left) and O (right) adsorption energies as a function of nanoparticle charge/Pd atom.

2.15 EEL spectroscopy at Si K edge

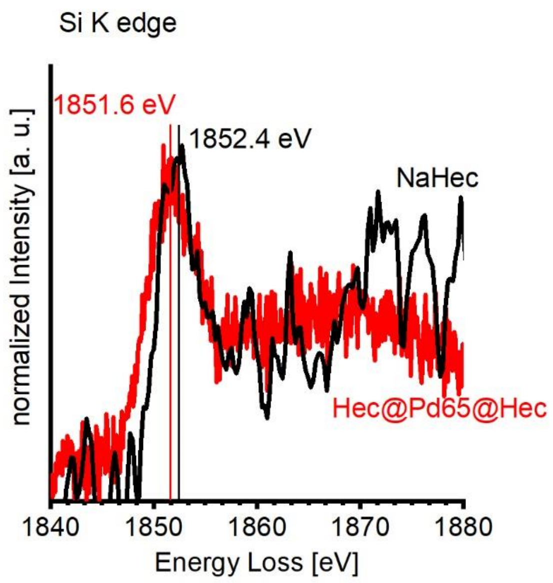

Figure S18. ELL spectra at the Si K edge of NaHec (black) and Hec@Pd65@Hec (red).

3 Author Contributions

Kevin Ament designed and performed the experiments, characterized the catalysts and evaluated the data. Kevin Ament and Josef Breu wrote the manuscript. Nicolas Köwitsch and Marc Armbrüster supported the evaluation of the conducted catalytic experiments and assisted with the writing of the manuscript. Dianwei Hou and Christopher J. Heard did the computational investigation and wrote this part of the manuscript. Thomas Götsch und Thomas Lunkenbein performed EELS. Jutta Kröhnert performed DRIFTS measurements and Annette Trunschke assisted with interpretation and commented on the manuscript.

4 References

[1] K. A. Flanagan, J. A. Sullivan, H. Müeller-Bunz, *Langmuir* **2007**, *23*, 12508-12520.
[2] M. Stöter, D. A. Kunz, M. Schmidt, D. Hirsemann, H. Kalo, B. Putz, J. Senker, J. Breu, *Langmuir* **2013**, *29*, 1280-1285.
[3] a) G. Kresse, J. Hafner, *Phys. Rev. B* **1993**, *47*, 558; b) G. Kresse, J. Hafner, *Phys. Rev. B* **1994**, *49*, 14251; c) G. Kresse, J. Furthmüller, *Computational Materials Science* **1996**, *6*, 15; d) G. Kresse, J. Furthmüller, *Phys. Rev. B* **1996**, *54*, 11169.
[4] P. E. Blöchl, *Phys. Rev. B* **1994**, *50*, 17953.
[5] J. P. Perdew, K. Burke, M. Ernzerhof, *Phys. Rev. Lett.* **1997**, *78*, 1396.
[6] S. Grimme, J. Antony, S. Ehrlich, H. Krieg, *The Journal of Chemical Physics* **2010**, *132*, 154104.
[7] S. Grimme, S. Ehrlich, L. Goerigk, *J. Comput. Chem.* **2011**, *32*, 1456.
[8] H. J. Monkhorst, J. D. Pack, *Phys. Rev. B* **1976**, *13*, 5188.
[9] M. Thommes, K. Kaneko, A. V. Neimark, J. P. Olivier, F. Rodriguez-Reinoso, J. Rouquerol, K. S. W. Sing, *Pure Appl. Chem.* **2015**, *87*, 1051-1069.
[10] a) C. J. Zhang, P. Hu, *Journal of the American Chemical Society* **2001**, *123*, 1166-1172; b) Z. Duan, G. Henkelman, *ACS Catalysis* **2014**, *4*, 3435-3443; c) N. M. Martin, M. Van den Bossche, H. Grönbeck, C. Hakanoglu, F. Zhang, T. Li, J. Gustafson, J. F. Weaver, E. Lundgren, *The Journal of Physical Chemistry C* **2014**, *118*, 1118-1128; d) Z. Hooshmand, D. Le, T. S. Rahman, *Surface Science* **2017**, *655*, 7-11.
